# Comorbidity Influences Multiple Aspects of Well-Being of Patients with Ischemic Heart Disease

**Published:** 2013-12-01

**Authors:** Shervin Assari, Maryam Moghani Lankarani, Khodabakhsh Ahmadi

**Affiliations:** 1 Department of Health Behavior and Health Education, School of Public Health, University of Michigan, Ann Arbor, Michigan, USA; 2 Center for Research on Ethnicity, Culture, and Health (CRECH), School of PublicHealth, University of Michigan, Ann Arbor, Michigan, USA; 3 Medicine and Health Promotion Institute, Tehran, IR Iran; 4 Universal Network for Health Information Dissemination and Exchange (UNHIDE), Tehran, IR Iran; 5 Behavioral Sciences Research Center, Baqiyatallah University of Medical Sciences, Tehran, IR Iran

**Keywords:** Coronary Artery Disease, Sleep, Sexual Behavior, Psychological Distress

## Abstract

**Background:**

Comorbidity is prevalent among patients with Ischemic Heart Disease (IHD) and may influence patients’ subjective and objective domains of well-being.

**Objectives:**

We aimed to investigate the associations between comorbidity and different measures of well-being (i.e. health related quality of life, psychological distress, sleep quality, and dyadic adjustment) among patients with IHD.

**Methods:**

In this cross-sectional study, 796 outpatients with documented IHD were enrolled from an outpatient cardiology clinic in 2006. Comorbidity (Ifudu index), quality of life (SF36), psychological distress (Hospital Anxiety Depression Scale; HADS), sleep quality (Pittsburg Sleep Quality Index; PSQI), and dyadic adjustment quality (Revised Dyadic Adjustment Scale; RDAS) were measured. Associations between comorbidity and different measures of well-being were determined.

**Results:**

Significant correlations were found between comorbidity score and all measures of well-being. Comorbidity score was correlated with physical quality of life (r = -0.471, P < 0.001), mental quality of life (r = -0.447, P < 0.001), psychological distress (r = 0.344, P < 0.001), sleep quality (r = 0.358, P < 0.001), and dyadic adjustment (r = -0.201, P < 0.001).

**Conclusions:**

This study showed a consistent pattern of associations between somatic comorbidities and multiple aspects of well-being among patients with IHD. Findings may increase cardiologists’ interest to identify and treat somatic conditions among IHD patients.

## 1. Background

Comorbidity refers to the coexistence of other chronic diseases in patients with an index disease (1). As most patients with chronic conditions also suffer from other health conditions, assessment of comorbidity plays an essential role in the care of patients with chronic conditions (2). Measurement of comorbidities and their link to other health outcomes is an essential part of assessment of patients with chronic medical conditions (3, 4).

Most but not all (5, 6) studies have suggested that medical comorbidity affects health and well-being (7-11), functional status, health related quality of life (HRQoL), disability (12), and mortality (13) of patients with chronic conditions. Some researchers believe that psychiatric comorbidities, but not medical comorbidities, are associated with well-being of patients with chronic medical conditions. In one study, for instance, psychiatric but not medical comorbidities were associated with lost productivity (14).

To date, most of our knowledge about the link between comorbidity and health and well-being (15-25) is limited to certain measures of health, and very few studies have ever assessed the association between somatic comorbidities and other aspects of patients’ well-being, such as sleep quality and dyadic relationship (26).

Ischemic Heart Disease (IHD) is one of the leading causes of death in the world (23). Among patients with IHD, comorbid health conditions profoundly affect morbidity (8) and mortality (1). Few studies, however, have focused on the association between somatic comorbidities and multiple aspects of morbidity among patients with IHD.

In the current study we aimed to investigate the associations between comorbidity and different aspects of morbidity among patients with IHD.

## 2. Patients and Methods

### 2.1. Study Design and Patients

This cross-sectional study was a part of a greater research project, which was conducted in 2006 at the outpatient clinic of the Baqiyatallah hospital, Tehran. Other reports have been published from the main study (27-30).

The current study was conducted on 796 consecutive patients with coronary artery disease (defined by a 70% stenosis of at least 1 major coronary artery, confirmed by angiography). Written informed consent was obtained from all patients. The Ethics Committee of Baqiyatallah University approved the study protocol.

The demographic data checklist included age, sex, marital status, family income, educational level, living place, and body mass index. HRQoL, psychological status, sleep quality, and dyadic adjustment quality were assessed, as well.

### 2.2. Comorbidity

Comorbidity was measured using the modified version of the Ifudu comorbidity index. Ifudu comorbidity index is a numerical self-reporting measure originally designed to evaluate comorbidities among the chronic renal patients. It evaluates the presence of 13 chronic illnesses. Each co-morbid condition is scored from 0 to 3 representing the absence of the disease and the presence of severe disease, respectively. The total comorbidity score is then calculated by summing up the scores for all of the 13 organ systems. The total score ranges from 0 to 39, with a higher score being indicative of a greater comorbidity (31).

In our modified version, we omitted the item related to IHD since it was the index disease in our study. We also omitted the item related to psychiatric disorders to cover somatic comorbidities only. The modified Ifudu index in this study consisted of 11 items and was scored from 1 to 33. Although this measure was originally developed for patients with chronic kidney disease, it has been repeatedly used in a wide range of chronic conditions, such as respiratory disease, blood disease, rheumatologic conditions, and IHD (32-35).

### 2.3. Psychological Distress

Psychological distress was assessed using the Hospital Anxiety Depression Scale (36), a translated version that had been previously validated for the Iranian population (37). The HADS contains 14 items on the symptoms of anxiety and depression. Each item is scored from 0 to 3, giving the maximum score of 42 for psychological distress (38-43).

### 2.4. Sleep Quality

Sleep quality over the month before the study was determined using the Pittsburg Sleep Quality Index (PSQI), which includes 7 items. The total score of this scale ranges from 0 to 21, with higher scores indicating worse sleep quality (44-47).

### 2.5. Dyadic Adjustment

All the study subjects completed a previously translated version (27, 48, 49) of the Revised Dyadic Adjustment Scale (RDAS) (50, 51). The RDAS consists of 14 items measuring the couples’ agreement on decisions and appropriate behavior, dyadic satisfaction, and dyadic cohesion. RDAS scores range from 0 to 69, with a low score being indicative of distress in dyadic adjustment. The scale provides a total score and 4 sub-scores including dyadic consensus (measures the degree to which the couple agrees on matters of importance to the relationship), affective expression (measures the degree of demonstrations of affection and sexual relationships), dyadic satisfaction (measures the degree to which the couple is satisfied with their relationship), and dyadic cohesion (measures the degree of closeness and shared activities experienced by the couple) (50, 51). The Cronbach's alpha was 0.802, 0.683, 0.779, 0.827, and 0.836 for the total score, dyadic consensus, affective expression, dyadic satisfaction, and dyadic cohesion, respectively.

### 2.6. HRQOL

The 36-item Medical Outcome Study Short Form Health Survey (SF-36) was used to measure the HRQOL (52). The SF-36 is a generic measure of HRQOL that contains eight subscales. These scales represent physical functioning, social functioning, role limitations due to physical health problems, role limitations due to emotional problems, mental health, vitality, bodily pain, and general health perceptions.

The subscale scores are transformed to a 0 - 100 scale, with higher scores indicating higher HRQOL. Physical and mental components of the eight scales were combined into a Physical Composite Score (PCS) and a Mental Composite Score (MCS) (53).

The Persian version of the SF-36 was used to ensure the scale’s face validity and maximize its acceptability among the Iranian participants (7, 32, 54-56). In this study, we used the total SF-36 score as the dependent variable; subscales and composite scores were not included. The SF-36 questionnaires were completed by the patients. Assistance for completion of the questionnaires was needed in a few cases.

### 2.7. Statistical Analysis

In this study, statistical analysis was performed using the SPSS-13 (SPSS Inc. IL) statistical software package. First, we checked the normal distribution of the variables using Kolmogorov-Smirnov test. Descriptive statistics were utilized for somatic comorbidities. In addition, the difference between male and female patients regarding comorbidity was assessed using independent samples t- test. In addition, the correlation between the comorbidity score and age, dyadic relationship quality, psychological factor, total PSQI score, total SF36 score and subscales was evaluated using a partial correlation test (age and gender controlled).

## 3. Results

### 3.1. Patients

The present study was conducted on 525 male (66.0%) and 271 female (34.0%) patients. The mean age of the subjects was 57.78 ± 11.52 years (range, 30 to 83).

### 3.2. Comorbidity

Mean (SD) comorbidity score was 2.98 ± 2.08. The most prevalent chronic conditions were lower back pain, spine or joint disorders (n = 481, 60.4%), non- ischemic heart disease (n = 363, 45.6%), musculoskeletal disorders (n = 295, 37.1%), and neurological disorders (n = 282, 35.4%) (Table 1). 

**Table 1.** Frequency of Various Different Comorbid Conditions among the 796 Iranian Patients with Documented Ischemic Heart Disease

Characteristics	N (%)
**Low back pain, spine or joint disorders**	
No	315 (39.6)
Yes	481 (60.4)
**Non-ischemic heart disease**	
No	433 (54.4)
Yes	363 (45.6)
**Musculoskeletal disorders**	
No	501 (62.9)
Yes	295 (37.1)
**Neurological disease**	
No	514 (64.6)
Yes	282 (35.4)
**Hematological disease**	
No	606 (76.1)
Yes	190 (23.9)
**Chronic respiratory disease**	
No	620 (77.9)
Yes	176 (22.1)
**Visual impairment**	
No	623 (78.3)
Yes	173 (21.7)
**Infections**	
No	644 (80.9)
Yes	152 (19.1)
**Urology diseases**	
No	647 (81.3)
Yes	149 (18.7)
**Liver, pancreas or biliary disease**	
No	701 (88.1)
Yes	95 (11.9)
**Peripheral vascular**	
No	778 (97.7)
Yes	18 (2.3)

### 3.3. Comorbidity and Socio-Demographic Variables

The study results revealed a significant and positive correlation between the comorbidity score and age (r = 0.146, P < 0.001). Moreover, the results of the T-test showed a significantly higher comorbidity score among the female patients (4.0 ± 2.0, 2.42 ± 1.87, P < 0.001), married patients (3.61 ± 2.23, 2.87 ± 2.0, P < 0.001), the patients with lower than diploma education levels (3.35 ± 2.0, 2.24 ± 1.82, P < 0.001), and those with lower than 400 US$ monthly income (3.37 ± 1.99, 2.79 ± 2.1, P < 0.001). However, comorbidity score did not show significant associations with living in urban area (P = 0.358) and BMI level (P = 0.138) (Table2).

**Table 2.** The Association between the Socio-Economic Characteristics and Comorbidity among 796 Patients with Ischemic Heart Disease[Table-fn fn6738]

Characteristics	N (%)	mean ± SD	*P value*
Gender			
Male	525 (66.0)	2.42 ± 1.87	< 0.001 ^a^
Female	271 (34.0)	4.0 ± 2.0	
**Marital Status**			
Married	680 (85.4)	2.87 ± 2.0	< 0.001 ^a^
Single, divorced, widow	116 (14.6)	3.61 ± 2.23	
**Education**			
Not completed high school	530 (66.6)	3.35 ± 2.0	< 0.001 ^a^
Completed high school	266 (33.4)	2.24 ± 1.82	
**Living place**			
Urban	727 (91.3)	2.96 ± 2.1	0.358 ^a^
Suburban	69 (8.7)	3.2 ± 1.88	
**Family income**			
Lover than 200$	260 (32.7)	2.79 ± 2.1	< 0.001 ^a^
Upper than 200$	536 (67.3)	3.37 ± 1.99	
**Body Mass Index**			
≤ 25	300 (37.7)	2.78 ± 1.96	0.138 ^b^
25 - 30	263 (36.8)	3.0 ± 2.18	
≥ 30	184 (23.1)	3.1 ± 2.0	

Abbreviations: SD, Standard Deviation;

^a^Independent samples test;

^b^One-way ANOVA

### 3.4. Comorbidity and Health Related Quality of Life

Based on partial correlation, significant negative correlations were found between the comorbidity score and SF36 total score (P < 0.001), physical function (P < 0.001), role limitations due to physical problems (P < 0.001), bodily pain (P < 0.001), social function (P = 0.004), general mental health (P < 0.001), role limitations due to emotional problems (P < 0.001), vitality (P < 0.001), general health perceptions (P < 0.001), PCS (P < 0.001), and MCS (P < 0.001).

### 3.5. Comorbidity and Psychological Distress

Partial correlation showed a significant positive correlation between comorbidity score and psychological distress (HADS total score) (P < 0.001) (Table 3). 

**Table 3.** Correlations between Comorbidity and Multiple Aspects of Well-Being among the 796 Iranian Patients with Ischemic Heart Disease[Table-fn fn6741]

	r	*P value*
**Quality of life (SF-36)**		
Physical function	-0.378	0.001
Role limitations	-0.308	0.001
Bodily pain	-0.353	0.001
Social function	-0.102	0.004
General mental health	-0.388	0.001
Role limitations due to emotional problem	-0.32	0.001
Vitality energy or fatigue	-0.383	0.001
General health perceptions	-0.405	0.001
Physical composite score	-0.471	0.001
Mental composite score	-0.447	0.001
Total score	-0.488	0.001
**Sleep quality (PSQI)**		
Total score	0.358	0.001
**Dyadic relationship (RDAS)**		
Total score	-0.201	< 0.001
Dyadic consensus	-0.103	0.009
Dyadic expression	-0.088	0.028
Dyadic satisfaction	-0.261	0.001
Dyadic cohesion	-0.105	0.008
**Psychological distress (HADS)**		
Total score	0.344	0.001

Abbreviations: RDAS, Revised Dyadic Adjustment Scale; PSQI, Pittsburg Sleep Quality Index; HADS, Hospital Anxiety Depression Scale Results are based on partial correlation tests. Age and gender have been controlled.

### 3.6. Comorbidity and Sleep Quality

Based on partial correlation, a significant positive correlation was found between the comorbidity score and PSQI total score (P < 0.001).

### 3.7. Comorbidity and Dyadic Adjustment

Partial correlation revealed significant negative correlations between the comorbidity score and RDAS total score (P < 0.001), RDAS consensus score (P = 0.009), RDAS dyadic expression score (P = 0.028), RDAS dyadic satisfaction score (P < 0.001), and RDAS dyadic cohesion score (P = 0.008).

## 4. Discussion

The present study confirmed the associations between somatic comorbidity and multiple aspects of well-being of the patients with IHD. Based on our findings, presence of somatic comorbidity is associated with poor quality of life, sleep quality, dyadic adjustment, and high psychological distress.

An association between higher somatic comorbid diseases and poor HRQoL was expected. This finding is in line with studies by Baumeister et al. (26) and Gijsen et al. (57) which suggested that comorbidity was associated with reduced function and quality of life among patients with a somatic index disease.

Our study also showed an association between comorbidity and poorer sleep quality, psychological wellbeing, and dyadic quality. The consistent associations between comorbidities and multiple aspects of well-being of the patients reflect the role of other chronic medical conditions on morbidity of IHD patients (26). The result of this study advocates for screening, diagnosis and treatment of other chronic medical conditions among patients with IHD.

Comorbid conditions are the rule rather than the exception, and affect almost all the health outcomes. As multiple aspects of well-being are important for the patients, an increase in attention to patients’ somatic comorbidities may improve patients’ well-being (26). Hence, physicians and other healthcare providers who wish to improve the subjective well-being of IHD patients may need to target somatic comorbidities in their management plan.

Typically, physicians and other healthcare providers mostly focus on the index disease, considering the index disease as the major source of morbidity. However, any medical decision for the patient is taken in the presence of other, often uncontrollable, medical conditions. Unfortunately, less has been discussed in the literature about the importance of incorporating medical comorbidities in decision making and care for chronic patients (58, 59). This is partly because comorbidity is mostly conceptualized as a confounder. However, medical comorbidities are of utmost importance in making decisions on treatment choices (58-60). Comorbidity influences the prognosis of index disease (2, 61-65).

We must acknowledge the possible limitations to the generalizability of the findings of the current study. We sampled our patients from a single center (large teaching hospitals) and, consequently, our results may not apply to other centers, especially with different conditions. In addition, comorbidity was measured using self-reported data. Thus, further researches should be conducted in this area. This study, however, covered a wide range of well-being measures. It is perhaps surprising that only a modest amount of research has been devoted to describing the prevalence and impact of comorbidity among patients with IHD (66, 67).

To conclude, this study provided evidence regarding modest associations between comorbidity and multiple aspects of well-being of the patients with IHD. The current findings are hoped to increase cardiologists' interest to diagnose and treat other medical conditions that IHD patients may have.
